# Circulating tumour cells and circulating cell-free DNA in patients with lung cancer: a comparison between thoracotomy and video-assisted thoracoscopic surgery

**DOI:** 10.1136/bmjresp-2021-000917

**Published:** 2021-09-07

**Authors:** Periklis Katopodis, Vladimir Anikin, Uday Kishore, Thomas Carter, Marcia Hall, Nizar Asadi, Andreas Polychronis, Emmanouil Karteris

**Affiliations:** 1 Biosciences, College of Health, Medicine and Life Sciences, Brunel University London, Uxbridge, UK; 2 Thoracic Surgery, Royal Brompton & Harefield NHS Foundation Trust, Harefield, UK; 3 Department of Oncology and Reconstructive Surgery, Sechenov First Moscow State Medical University, Moscow, Russia; 4 Mount Vernon Cancer Centre, Northwood, UK; 5 Royal Brompton and Harefield NHS Trust, London, UK

**Keywords:** thoracic surgery, non-small cell lung cancer, lung cancer

## Abstract

**Introduction:**

The type of lung cancer surgery impacts on tumour manipulation during surgery and may drive dissemination of cancer cells into the vasculature, thus facilitating metastatic spread. The aim of this study was to investigate the impact of surgically induced trauma using peripheral blood from preoperative and postoperative patients with non-small cell lung cancer (NSCLC) undergoing thoracotomy or video-assisted thoracoscopic surgery (VATS) resection.

**Methods:**

Imaging flow cytometry was used to measure circulating cancer-associated cells (CCs). Circulating cell-free DNA (ccfDNA) isolation was performed using Promega dsDNA HS Assay Kit. DNA integrity measurements were calculated by the ALU247 to ALU115 ratio and cytokine levels measured using the Luminex screening assay.

**Results:**

CCs were increased in postoperative blood samples in 54 patients with NSCLC. Patients who underwent thoracotomy instead of VATS had higher numbers of EpCAM (p=0.004) and PanCK-labelled (p=0.03) CCs postoperatively. ccfDNA and DNA integrity index were also significantly increased in postoperative samples (p=0.0009 and p=0.04), with concomitant increase in interleukin 6 and interleukin 10 levels in the same cohorts (p=0.0004 and p=0.034, respectively).

**Conclusions:**

In this study we have shown the potential clinical utility of several biomarkers from liquid biopsies to guide perioperative management, as well as provide a snapshot of the type of surgical resection in terms of circulating tumour cell release. Obtaining reliable readouts from blood can provide crucial information for disease progression, as well as being of prognostic value monitoring patients’ response to treatment.

**Key messages**

- Surgery has an impact on the quantities of cancer-associated circulating cells (CCs) and circulating cell-free DNA (ccfDNA) and DNA integrity indices in patients with non-small cell lung cancer (NSCLC).
- We demonstrate the clinical potential of multiple liquid biomarkers readouts obtained from perioperative blood samples in patients with NSCLC.
- The study provides evidence of the different types of potential circulating biomarkers (cancer-associated CC, ccfDNA and DNA integrity in patients with operable lung cancer), adding vital data to the growing body of literature by studying multiple readouts in parallel from paired, clinical liquid biopsies.

## Introduction

During tumour development, individual cancer cells independently acquire genetic and epigenetic alterations via adaptation-related mutagenesis forming heterogeneous tumour cell populations.^1^ Tumour cells that may eventually acquire metastatic potential migrate away from the primary tumour via the bloodstream or lymphatic system. Cancer-associated circulating cells (CCs), or circulating tumour cells (CTCs), are malignant cells present in the bloodstream that originate either from the primary tumour or a metastatic deposit. They often possess tumour-specific antigenic and/or genetic signatures through which they are identifiable, despite their scarcity in peripheral blood compared with peripheral blood mononuclear cells.^2 3^ Although more commonly referred to as CTCs, these cells are a heterogeneous group, and limitations still exist in characterising them in detail. In view of this, using the nomenclature ‘non-haematopoietic cancer-related circulating cells’ may currently more accurately reflect what these cells are.

Identification and characterisation of CCs is a promising tool which could facilitate earlier diagnosis of malignancy and provide real-time information on treatment efficacy and therefore prognosis. Clinically, CCs have been used for early detection of a number of cancers, including non-small cell lung cancer (NSCLC).^4^ A number of studies have reported a positive association between the number of CCs and the clinical stage of the tumour or the existence of distant metastases.^5–7^ CCs are often recognised using high-definition imaging combined with fluorescent digital scanners or size exclusion. These techniques also allow in situ molecular assays to be conducted, identifying genomic mutations on a single-cell basis.^8 9^

In addition to CCs, tumour DNA is also present in peripheral blood, in part due to the high apoptotic rate within the tumour microenvironment, which results in the release of DNA from apoptotic cells into the bloodstream, where it is known as circulating cell-free DNA (ccfDNA). When ccfDNA originates from apoptotic cells, it is usually found to be in 160–200 bp fragments, in contrast to ccfDNA from necrotic cells which often varies in length.^10–12^ In both scenarios (ie, apoptosis or necrosis), ccfDNA concentration in the plasma or serum is increased in patients with cancer when compared with healthy control subjects, a difference also observed in CC numbers.^13 14^

Circulating tumour DNA (ctDNA) is the portion of ccfDNA specifically derived from cancer cells and has been estimated to range from 10% to 90% of the total ccfDNA in patients with cancer. DNA integrity measurement is one of the many ways to exploit further the clinical utility of the plasma ccfDNA. DNA integrity is generally calculated as the ratio of the concentration of longer to shorter DNA fragments (ie, ALU247:ALU115).^15^ This ratio represents the longer fragments generated more randomly (ie, by necrosis) compared with the smaller fragments (~180 bp) produced by physiological apoptosis.^16^ As the tumour progresses, the DNA integrity index is expected to be higher since the cell death of tumour cells becomes a predominantly necrotic process compared with the programmed apoptotic cell death of normal cells.^17^ Detection of these longer ctDNA fragments and quantification of their relative abundance in plasma compared with short ccfDNA fragments and the calculation of a DNA integrity index have been explored as a potential cancer monitoring technique. To date, a number of studies have successfully validated these readouts in a variety of tumour types, including NSCLC.^16 17^

While surgical resection is the gold standard curative treatment for NSCLC, less than 20% of patients present with operable disease (National Cancer Registration and Analysis Service; ncin.org.uk). Even in patients in whom resection is possible, recurrence rates remain high, with 5-year survival rates of 56% in stage I disease and 34% in stage II disease (Office for National Statistics; ons.gov.uk). Thoracotomy has been the standard surgical option for tumour resection, but video-assisted thoracoscopic surgery (VATS) is increasingly being chosen.^18^ Although technically challenging, VATS procedures present a less invasive approach with a reduced risk of infection and shorter recovery times.^19^ This minimally invasive technique may also affect the dissemination of cancer cells into the vasculature impacting metastatic spread. To the best of our knowledge, this is the first study to compare multiple readouts (eg, CTCs and ctDNA) in parallel from paired, clinical liquid biopsies.

Lung tumour manipulation during surgery has been found to correlate with the number of CCs found within the pulmonary vein.^20 21^ However, the comparison of open versus minimally invasive approaches on the egress of tumour cells into circulating blood has not been investigated fully in the clinical setting. The phenomenon of increase of CTC in blood flow in relation to tumour manipulation has been confirmed experimentally. For example, in a preclinical study using a syngeneic in vivo model of primary renal tumours in which surgical resection of the primary tumours led to enhanced dissemination of the cancer, increased incidence of regional node metastasis and an increase in the number of CCs in the venous circulation.^22 23^ Of note, it has been shown that interventions including biopsy, surgery, laser treatment or even application of pressure may increase CCs in mice that were inoculated with melanoma or breast cancer cells.^24^

The aim of this study was to use preoperative and postoperative peripheral blood samples from patients with NSCLC undergoing thoracotomy or VATS resection to study the impact of surgery on levels of cancer-associated CCs and ccfDNA, DNA integrity indices and inflammatory cytokine profiles.

## Materials and methods

### Blood samples

Blood samples from patients undergoing surgical resection for NSCLC were collected at the Royal Brompton & Harefield NHS Trust and the East and North Herts NHS Trust, as part of the Circulating Cells In Advanced Cancer (CICATRIx; Integrated Research Approval System (IRAS) ID: 198179) clinical study. Samples were collected 1 day before the operation and 2–4 days after, including normal healthy volunteers (not undergoing surgery) as controls. Informed consent for participation was obtained from all donors. Blood samples were collected in ccfDNA collection Roche tubes (Roche Diagnostics, Mannheim, Germany) as previously described.^25^ Two tubes of 8 mL of whole blood were collected by venepuncture from each individual for image flow cytometry analyses and plasma extraction. The tube was centrifuged for 20 min at 2000× *g*, followed by removal of the plasma layer without disturbing the buffy coat and the red blood cell sediment. Each sample was spun again for 10 min at 2000× *g*, then plasma extracted and stored in −80°C until extraction of ccfDNA.

The flow of the study consisted of patients’ consent to be included in the study, admission to Harefield Hospital and confirmed diagnosis of lung cancer. Exclusion criteria were refusal to participate in the study and previous malignancies. The main evaluation items were CCs, ccfDNA and ctDNA, and the accessory evaluation items were clinical examination, spirometry, CT scan, positron emission tomography-CT scan and additional investigations on individual indications.

### ccfDNA isolation

ccfDNA was extracted using the Maxwell RSC ccfDNA Plasma Kit (RSC; Promega, Leiden, The Netherlands). A total of 50 µL of ccfDNA were extracted from 1000 µL of plasma according to the manufacturer’s protocol. All the samples were quantified by Quantus Fluorometer and/or Qubit dsDNA HS Assay Kit (ThermoFisher Scientific, Aalst, Belgium) using Qubit assay tubes (cat. Q32856) and following the manufacturer’s protocol.

### qPCR of ALU repeat elements

Quantification of DNA fragments was performed by qPCR (QuantStudio 7, ThermoFisher Scientific), which quantified and amplified the shorter and longer fragments (ALU repeats).^10 17^ The quantitative values from the 115 bp primers represent the total level of ccfDNA (ng/mL), while the ratio of longer to shorter fragments (ALU247:ALU115) indicates the integrity of ccfDNA in each sample. The sequences of the ALU115 and ALU247 primers were as follows: ALU115 forward 5′-CCTGAGGTCAGGAGTTCGAG-3′ and reverse 5′- CCCGAGTAGCTGGGATTACA-3′; ALU247 forward 5′-GTGGCTCACGCCTGTAATC-3′ and reverse 5′-CAGGCTGGAGTGCAGTGG-3′. The absolute amount of ccfDNA in each sample was determined by a standard curve using 10-fold dilutions (0.01–10 000 ng/mL) of human genomic DNA (cat. G3041; Promega, Madison, Wisconsin, USA). The reaction mixture for each qPCR contained 1 µL of DNA template, 10 µL SYBR Green PCR Master Mix (cat. 4309155; ThermoFisher), 0.5 µL of each primer (10 µM) and 8 µL of double distilled nuclease-free water (cat. AM9937; Invitrogen) in a total reaction volume of 20 µL with 95°C for 10 min, followed by 35 cycles of 95°C for 15 s and annealing at 60°C for 1 min. All assays were carried out in triplicates and a non-template control was included in every run. Concentration of the unknown samples was determined using the standard curves that were drawn for the genomic DNA for both ALU115 and ALU247.

### CC detection: ImageStream

Characterisation and enumeration of CCs took place using ImageStream as previously described.^26^ All the data files were then analysed on the IDEAS software. Quantification was calculated as positive cells per 1 mL. After every run, 20 µL of the cell suspension were dispersed on a microscope slide to verify the staining and characterise the positive cells further using the Leica DM4000 (Leica Microsystems).

### Magnetic Luminex screening assay

Cytokine levels were measured using a custom kit of a human cytokine/chemokine magnetic bead panel (cat. HTH17MAG-14K; EMD Millipore) as previously described.^27^

### Statistical analysis

Statistical analyses were performed by GraphPad Prism V.8 software. Continuous variables for the paired samples such as in CCs, ccfDNA, cytokines and ALU repeats were compared using the Wilcoxon matched paired test or unpaired t-test, where needed (thoracotomy preoperatively vs VATS preoperatively). Statistical significance was considered at p<0.05. Bars in the graphs indicate mean value with SEM.

## Results

### Patient characteristics

Fifty-four patients with NSCLC (23 male, 31 female) underwent surgical resection: 38 had adenocarcinoma (AC) and 16 had squamous cell carcinoma (SCC). Of the patients, 35 were stage I, 10 were stage II, 8 were stage III and 1 was stage IV. Of the patients, 12 underwent thoracotomy and 42 underwent VATS. The surgeries were performed in the thoracic surgical department of Harefield Hospital by one of the consultant chest surgeons according to discussion on a multidisciplinary team meeting. The average tumour size was 30.8 mm (±20.6), and 17 out of 54 patients had lymphovascular invasion and 8 out of 54 had lymph node involvement. Blood samples from the entire cohort were analysed for the presence of CCs preoperatively and postoperatively. Plasma samples for ccfDNA were available for 46 patients (85%), while 19 patients (35%) possessed samples suitable for cytokine multiplex assay analyses. Full demographics are shown in table 1.

**Table 1:** Demographic and clinical characteristics of patients with NSCLC used in each of the main analyses: CCs, ccfDNA and multiplex assay

CCs		ccfDNA		Multiplex assay	
Characteristics		Characteristics		Characteristics	
Total	54	Total	46	Total	19
Mean age	67.3 (±9.3)	Mean age	68 (±8.96)	Mean age	66 (±9.7)
Male/female (%)	23 (43)/31 (57)	Male/female (%)	19 (38)/27 (62)	Male/female (%)	6 (31)/13 (68)
**Pathology (n=54), n (%)**		**Pathology, n (%)**		**Pathology, n (%)**	
AC	38 (69)	AC	33 (72)	AC	15 (68)
SCC	16 (31)	SCC	13 (28)	SCC	4 (32)
Control	10	Control	8	Control	1
**Staging (n=54), n (%)**		**Staging (n=46), n (%)**		**Staging, n (%)**	
I	35 (65)	I	29 (63)	I	12 (63.5)
II	10 (18.5)	II	9 (19.5)	II	6 (31.5)
ΙΙΙ	8 (15)	ΙΙΙ	7 (15)	ΙΙΙ	1 (5)
IV	1 (1.5)	IV	1 (2.5)	IV	0 (0)
**Operation (n=54), n (%)**		**Operation, n (%)**		**Operation, n (%)**	
Thoracotomy	12 (23)	Thoracotomy	9 (20)	Thoracotomy	2 (10)
Male/female (%)	7 (58)/5 (42)	Male/female (%)	4 (44)/5 (56)	Μale/female (%)	0 (0)/2 (100)
VATS	42 (77)	VATS	37 (80)	VATS	17 (90)
Male/female (%)	16 (38)/26 (62)	Male/female (%)	15 (40)/22 (60)	Male/female (%)	6 (35)/11 (65)

AC, adenocarcinoma; ccfDNA, circulating cell-free DNA; CCs, circulating cells; NSCLC, non-small cell lung cancer; SCC, squamous cell carcinoma; VATS, video-assisted thoracoscopic surgery.

### Expression of CCs in preoperative versus postoperative patients with NSCLC

**Figure 1:**
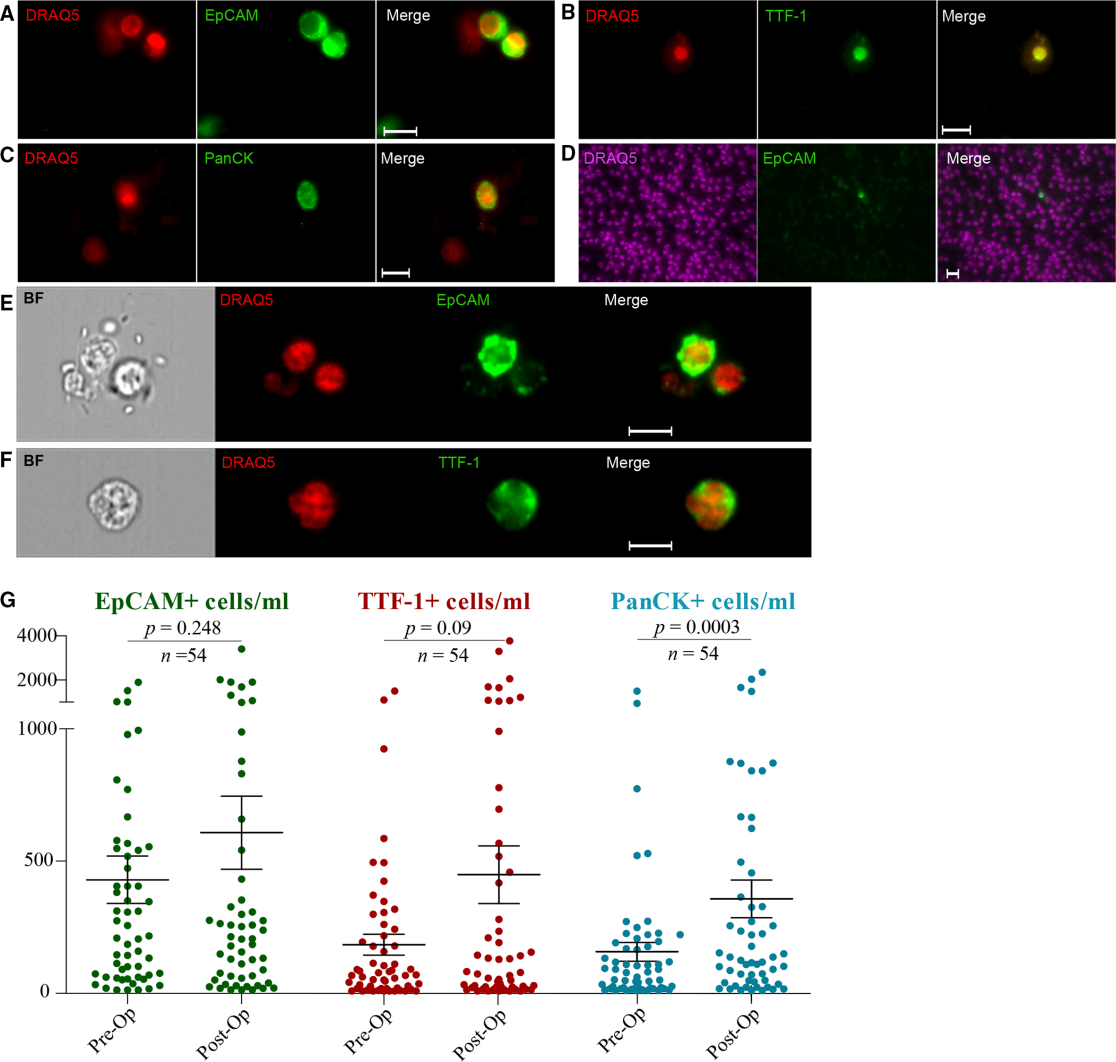
Representative images of immunofluorescence staining of patients’ blood sample on Leica DM4000 (scale bar 10 µm): (A) Epithelial cell adhesion molecule, EpCAM^+^ (two positive cells presented; stained green); the same cells stained with the DNA-specific marker DRAQ5 (1,5-bis{[2-(di-methylamino) ethyl]amino}-4, 8-dihydroxyanthracene-9,10-dione, red); (B) TTF-1^+^ (one positive cell); and (C) Pan-cytokeratin, PanCK^+^ (one positive cell). (D) Example of an EpCAM^+^ cell in a 20 µL sample (scale bar for Leica images: 20 µm). ImageStream cell captures of (E) EpCAM^+^ and EpCAM^−^ cells and (F) Transcription Termination Factor 1, TTF-1^+^ cell (scale bar for ImageStream images: 10 µm). All samples were stained with the nuclear marker DRAQ5 (red, purple). (G) EpCAM^+^, TTF-1^+^ and PanCK^+^/CD45^−^ cells in preoperative and postoperative patients. Most of the patients showed elevated levels of detectable CCs following surgery. All paired groups were analysed with Wilcoxon signed-rank test and the mean values and SEM bar are presented in all scatter plots. BF, bright field; CCs, cancer associated circulating cells.

Fluorescent microscopy and image flow cytometry were used to enumerate and characterise CCs in peripheral blood, using established antibody-based techniques as previously described by our laboratory^26^ (figure 1A–F, online supplemental file 1).

10.1136/bmjresp-2021-000917.supp1 Supplementary data

Each of the three chosen CC markers (ie, EpCAM, PanCK and TTF-1) was analysed separately with preoperative samples compared with postoperative ones (n=54). We observed a statistically significant increase in the number of PanCK^+^ cells (mean values: preoperative=151, postoperative=361 cells; p*=*0.0003), while a trend towards an increase was observed for EpCAM (mean values: preoperative=418, postoperative=625; p*=*0.24) and TTF-1 (mean values: preoperative=181, postoperative=464 cells; p*=*0.09) (figure 1G).

**Figure 2:**
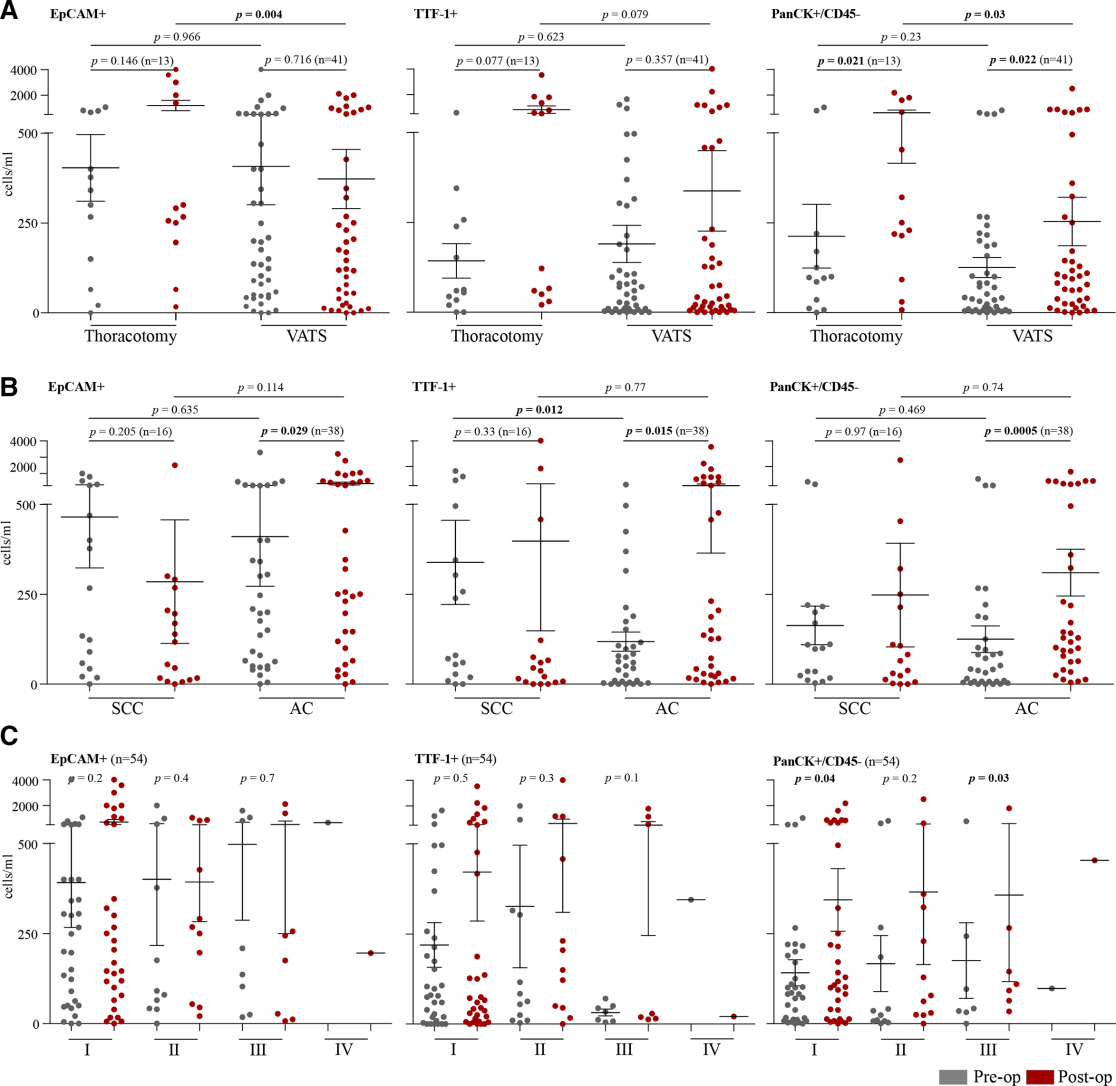
Subgroup analysis of preoperative and postoperative numbers of CC marker positive cells, i.e. Epithelial cell adhesion molecule, Transcription Termination Factor 1, and Pan-cytokeratin (EpCAM, TTF-1, PanCK). Cell numbers were analysed by (A) surgical approach (thoracotomy vs VATS); (B) NSCLC histotype (SCC vs AC); and (C) tumour stage (I–IV). All paired groups were analysed with Wilcoxon signed-rank test and the unpaired groups with an unpaired t-test. Mean values and SEM bars are presented in all scatter plots. AC, adenocarcinoma; CC, circulating cells; NSCLC, non-small cell lung cancer; SCC, squamous cell carcinoma; VATS, video-assisted thoracoscopic surgery.

Subgroup analysis was then conducted on preoperative/postoperative paired samples for each of the three studied CC markers, with analysis performed based on (1) surgical procedure (VATS vs open thoracotomy), (2) histology (AC vs SCC) and (3) postoperative tumour stage (I–IV), with results shown in figure 2 and online supplemental file 2.

10.1136/bmjresp-2021-000917.supp2 Supplementary data

CC levels for each of the studied markers were first compared between patients undergoing thoracotomy and VATS (figure 2A). A trend towards increased EpCAM^+^ CCs postoperatively was observed in thoracotomy patients (mean values: preoperative=403, postoperative=1200; p*=*0.146), with minimal change in EpCAM^+^ CCs observed following VATS (mean values: preoperative=412, postoperative=386; p*=*0.716). When comparing surgical method directly, a significant difference was seen in the number of postoperative EpCAM^+^ CCs between thoracotomy (1200 cells/mL, n=13) and VATS (386 cells/mL, n=41) (p=0.004). TTF-1^+^ CCs were found to increase postoperatively in all patients regardless of surgery, while the mean number of postoperative TTF-1^+^ CCs was higher in thoracotomy (825 cells/mL, n*=*13) versus VATS (353 cells/mL, n*=*41) patients; this was non-statistically significant (p*=*0.079). PanCK^+^/CD45^−^ CCs were found to significantly increase postoperatively in both thoracotomy patients (mean values: preoperative=212, postoperative=618; p*=*0.021) and VATS patients (mean values: preoperative=127, postoperative=259; p*=*0.022), while in direct comparison the number of postoperative PanCK^+^/CD45^−^ CCs was significantly higher (p*=*0.03) in thoracotomy patients (618 cells/mL, n*=*13) compared with those who had VATS (259 cells/mL, n*=*41).

Tumour-associated CCs levels were then compared between patients with AC and SCC (figure 2B). For postoperative patients with AC (n=38), the number of CCs increased significantly following surgery for all three markers (EpCAM^+^, p*=*0.029; TTF-1^+^, p*=*0.015; PanCK^+^, p*=*0.0005). For patients with SCC (n*=*10), no significant difference was observed between preoperative and postoperative samples.

Finally, the levels of CCs were compared between tumour stages (I–IV; figure 2C). For patients with stage I disease (n*=*35), the number of postoperative CCs was higher for all three CC markers studied, with significant differences seen in PanCK^+^ (p*=*0.04) CCs. In stage II disease (n*=*10) no significant difference was observed, whereas in stage III (n=8) a significant increase in PanCK^+^ cells was observed (p=0.03); the single stage IV patient does not allow comparison.

### Changes in ccfDNA and circulating cytokine levels following surgical trauma

**Figure 3:**
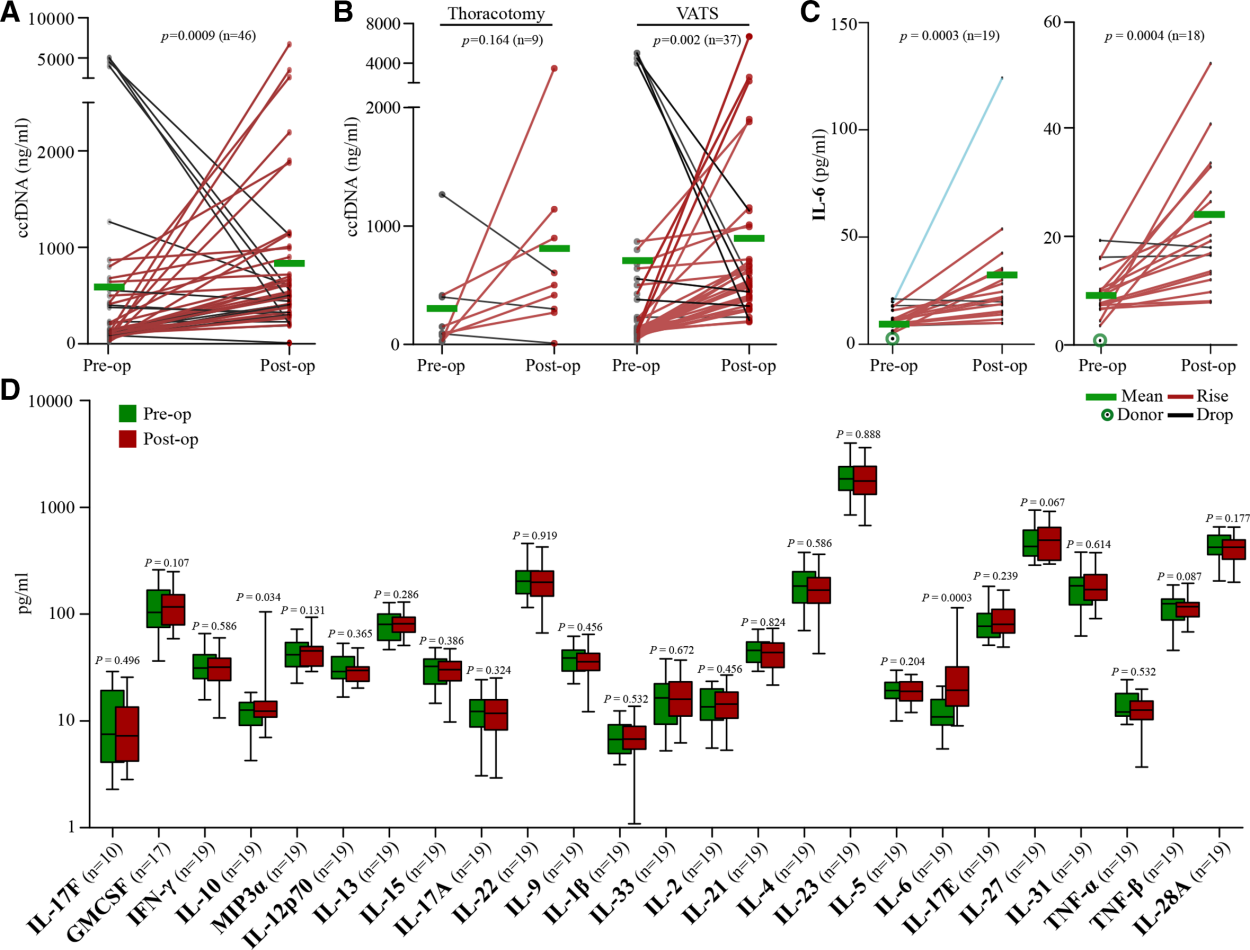
(A) Concentration of ccfDNA in preoperative and postoperative paired patient samples. (B) Comparison of ccfDNA concentration in patients undergoing thoracotomy and VATS. (C) IL-6 levels before and after surgery, with inset data excluding the outlier (blue). (D) Multiplex cytokine array analysis in plasma from preoperative and postoperative patients. Interleukin-6, IL-6 (p=0.0003) and IL-10 (p=0.034) were the only chemokines significantly upregulated in postoperative compared with preoperative samples. None of the other studied cytokines demonstrated significant changes in relation to surgery. All paired groups were analysed with Wilcoxon signed-rank test and the mean values and SEM bars are presented in all bar plots. ccfDNA, circulating cell-free DNA; IL, interleukin; VATS, video-assisted thoracoscopic surgery. GMCSF, granulocyte-macrophage colony-stimulating factor; IFN-γ, interferon gamma; MIP3α, macrophage inflammatory protein-3 alpha; ΤNFα, tumour necrosis factor alpha.

Using samples from the same patients, ccfDNA and inflammatory cytokine levels were measured (figure 3). Preoperative and postoperative ccfDNA levels were measured and the types of surgery compared. For all patients (n=46), the mean preoperative ccfDNA concentration was 624.7 ng/mL (range: 20–5000 ng/mL), compared with 863 ng/mL (range 10–6698) in postoperative samples (p*=*0.0009) (figure 3A). For patients who underwent thoracotomy (n*=*9), the mean preoperative ccfDNA was 283 ng/mL (range 24–1266 ng/mL) and the postoperative ccfDNA was 846 ng/mL (range 10–3480 ng/mL; p=0.16), while for those undergoing VATS (n*=*37) the mean preoperative ccfDNA was 707 ng/mL (range 20–5000 ng/mL) and the postoperative ccfDNA was 867 ng/mL (range: 190–6698 ng/mL; p=0.002) (figure 3B).

To measure preoperative and postoperative cytokine levels, a multiplex assay was performed demonstrating elevated interleukin (IL)-6 and to a lesser extent IL-10 levels postoperatively compared with baseline preoperative samples (p=0.0003 and p=0.034, respectively; left half of figure 3C, D). This result remained significant even with the exclusion of a single outlier (p=0.0004) (right half of figure 3C).

**Figure 4:**
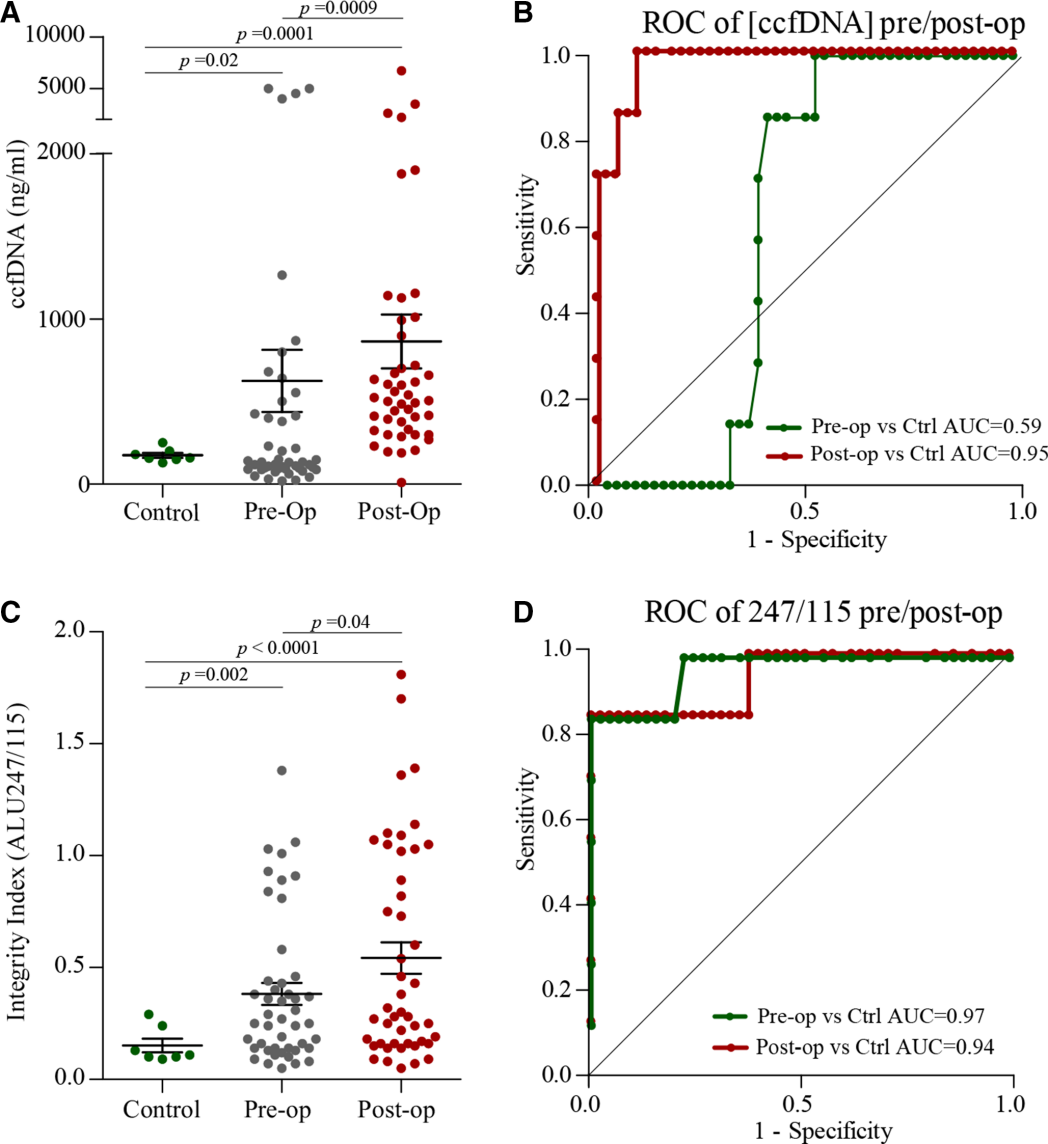
(A) Concentration of ccfDNA in preoperative and postoperative samples. (B) Receiver operating characteristic (ROC) curves for ccfDNA indicate that preoperative patients versus control (Ctrl) had an AUC of 0.59 (95% CI 0.45 to 0.74) and postoperative patients versus control had an AUC of 0.95 (95% CI 0.90 to 1.01). (C) DNA integrity index (ALU247:ALU115 ratio) in preoperative and postoperative samples. (D) Ratio of ALU247 to ALU115 concentration had high sensitivity and specificity in preoperative and postoperative analyses compared with controls, with an AUC of 0.97 (95% CI 0.91 to 1.02) and postoperative patients an AUC value of 0.94 (95% CI 0.84 to 1.04). AUC, area under the curve; ccfDNA, circulating cell-free DNA.

In addition to increasing postoperatively (figure 4A), ccfDNA was also significantly higher in patients with cancer both preoperatively (p*=*0.02) and postoperatively (p*=*0.0001) when compared with controls (figure 4A). The ROC (receiver operating characteristic) curve of ccfDNA for discriminating preoperative patients (n*=*46) from healthy individuals had an area under the curve (AUC) value of 0.59 (95% CI 0.45 to 0.74) and postoperative patients an AUC value of 0.95 (95% CI 0.9 to 1.01) (figure 4B).

To further characterise the relevance of elevated ccfDNA postoperatively and compared with healthy volunteer controls, data were further interrogated by measuring the DNA integrity index (online supplemental file 3). The ALU247 to ALU115 ratio was significantly elevated postoperatively (p*=*0.04) (figure 4C). Similarly to ccfDNA, both preoperative and postoperative ratios were also significantly increased compared with controls (p*=*0.002 and p*<*0.0001, respectively). The ROC curve of serum DNA integrity for discriminating preoperative patients with NSCLC (n*=*46) from healthy individuals had an AUC value of 0.97 (95% CI 0.91 to 1.02) and postoperative patients an AUC value of 0.94 (95% CI 0.84 to 1.04) (figure 4D).

10.1136/bmjresp-2021-000917.supp3 Supplementary data

## Discussion

In this study, we set out to investigate the effect of surgery on peripheral blood tumour-associated CCs and ccfDNA/ctDNA levels in patients with NSCLC. Our data suggest that CC levels are elevated postoperatively in patients of all groups when stained with epithelial, cytokeratin or TTF-1 markers, reaching statistical significance with the use of PanCK marker. To the best of our knowledge, this is the first study documented in patients with NSCLC and corroborates previous findings in patients undergoing surgery for hepatic metastases from colorectal cancer, where a substantial number of CTCs were detected intraoperatively when compared with their preoperative status.^28^ Following identification of CCs, we sought to establish whether there were differences between the surgical approach, tumour stage and histological subtype. Here we demonstrate that patients who underwent thoracotomy had higher numbers of EpCAM^+^ and PanCK^+^ cells postoperatively when compared with patients who underwent VATS.

Due to the larger incision and manual manipulation with thoracotomy, there is a higher likelihood of epithelial cells making their way into the circulation, which could explain why the mean number of PanCK^+^ (preoperative=212 cells; postoperative=618 cells), EpCAM^+^ (preoperative=403 cells; postoperative=1200 cells) and TTF-1^+^ (preoperative=142 cells; postoperative=825 cells) was higher after thoracotomy compared with VATS. Limitations of this study were the inclusion of one patient who was found to be stage IV postoperatively, where surgery may not necessarily impact CC/ctDNA levels, and the absence of benign surgical patients to act as controls. Further work to include a benign cohort and analyse early stage (I and II) and advanced stage (III and IV) patients with NSCLC separately is underway.

The significant increase (3.8-fold) in ccfDNA following tumour resection shown here corroborates findings from previous studies of patients with colorectal cancer undergoing elective surgery, where elevated levels of ccfDNA persisted for up to 4 weeks.^29^ The elevation of the DNA integrity index postoperatively represents an increase in the longer fragments of ccfDNA following surgical damage, likely to represent DNA from malignant cells (ctDNA); it is in line with other published data. These results corroborate the hypothesis that surgical trauma causes proportionate rises in ccfDNA.^30^

These results are also consistent with previous studies demonstrating the diagnostic potential of this measurement for other malignancies such as endometrial and breast cancer.^16 31 32^ It supports the hypothesis that surgical trauma leads to higher levels of ccfDNA and ctDNA. It has been previously argued that excess ccfDNA released postoperatively could hamper the detection of ctDNA; however, this does not appear to be the case in this study.^29^

Future work aims to validate these findings in a larger cohort of patients with NSCLC with serial blood samples drawn over a longer postoperative time to determine whether the levels of ccfDNA or ctDNA eventually fall below the preoperative levels. Correlation with the presence or absence of lymphovascular space invasion and hopefully tissue mutational status is also planned. Arguably showing identical mutational analyses of the CC±ctDNA with the same patient’s tissue would represent definitive evidence for the presence/absence of increased ctDNA in relation to thoracic surgery. Isolation of peripheral CTCs and subsequent sequence analysis are already planned and will provide further insight into the mutational landscape of these cells. Moreover, previous studies have identified CTCs based on size.^33 34^ In our study, after fluorescent microscopy analyses, the cells appeared to have similar sizes as leucocytes (10–12 µM; data not shown). In this study, enumeration and characterisation of cells were based on the positiveness of the antibodies used rather than size exclusion. Future studies using devices that isolate CTCs based on size (eg, Parsortix) can be used to potentially isolate CTCs from patients with NSCLC.^35 36^

Inflammatory changes following surgery are well documented.^37^ We detected the expected significant increase in IL-6 and IL-10 levels postoperatively when compared with matched preoperative samples. There is some evidence to suggest that certain laparoscopic procedures have been associated with lower cytokine levels when compared with their open counterparts.^37 38^ IL-6 is of particular interest, given that its release has been linked with postoperative systemic inflammatory response syndrome.^39^ Unfortunately, the majority of cytokine samples (18 out of 20) in this series were from patients undergoing VATS, so confirmation of this could not be made here. Future work should concentrate on measuring IL-6 levels in a larger population and stratifying the cohorts into those undergoing VATS versus thoracotomy. Despite the inherent flaw, it is clear that IL-6 can be a very useful marker of surgically induced trauma. IL-10 (also elevated postoperatively) is an anti-inflammatory cytokine which is activated by IL-6. IL-10 downregulates proinflammatory cytokines such as ΤNFα, IL-1β, IL-12 and IFNγ.^40–42^ It is therefore no surprise that none of the other cytokines studied had significant changes in their expression following surgery.

Although the comparative randomised trials (VATS vs thoracotomy in patients with NSCLC) are small, most agree that VATS is the preferable option with respect to complications and length of hospital stay.^39^ A meta-analysis also confirms a lower incidence of recurrence and an improved 5-year mortality rate for patients with early-stage NSCLC undergoing VATS.^43^ For example, among 100 patients with stage IA NSCLC, the overall 5-year survival rates postoperatively were 85% and 90% in the thoracotomy and VATS groups, respectively.^44^ Plans to consolidate this finding would require a larger scale randomised trial; the exploration of circulating biomarkers, as described here, postoperatively in such a trial could identify patients with better long-term outcomes.

## Concluding remarks

In addition to the expected increase in inflammatory cytokine levels, surgical trauma appears to cause surges in CCs in patients undergoing surgery for NSCLC, along with concomitant increases in ccfDNA and ctDNA. The potential clinical utility of several biomarkers from liquid biopsies to potentially monitor disease burden perioperatively includes more accurate prognostication for patients as well as guiding the role of adjuvant therapy (chemotherapy and radiotherapy). Blood-based biomarkers for postoperative surveillance could reduce the reliance on frequent imaging, improving the quality of life of these patients.^45^ This is of particular interest given that a smaller number of CTCs were detected in patients undergoing VATS when compared with thoracotomy. Our preliminary findings demonstrate some advantages of VATS over thoracotomy, but further investigations would be required to understand if there is a definitive link to surgical manipulation. Follow-up trials are required to correlate the levels of inflammatory cytokines, ccfDNA, ctDNA and/or CCs with progression-free survival and disease relapse and response to systemic treatment.

## Data Availability

Data are available upon reasonable request.
