# Supplementary figures and images for: Circulating tumour cells and circulating cell-free DNA in patients with lung cancer: a comparison between thoracotomy and video-assisted thoracoscopic surgery
Source: BMJ Open Respir Res. 2021 Sep 7;8(1):e000917. doi: 10.1136/bmjresp-2021-000917 (PMC8424856; doi:10.1136/bmjresp-2021-000917)

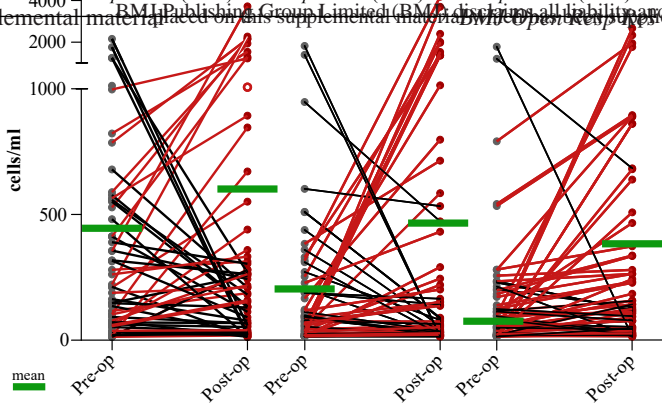

Supplement: Supplementary data [file bmjresp-2021-000917supp001.pdf]

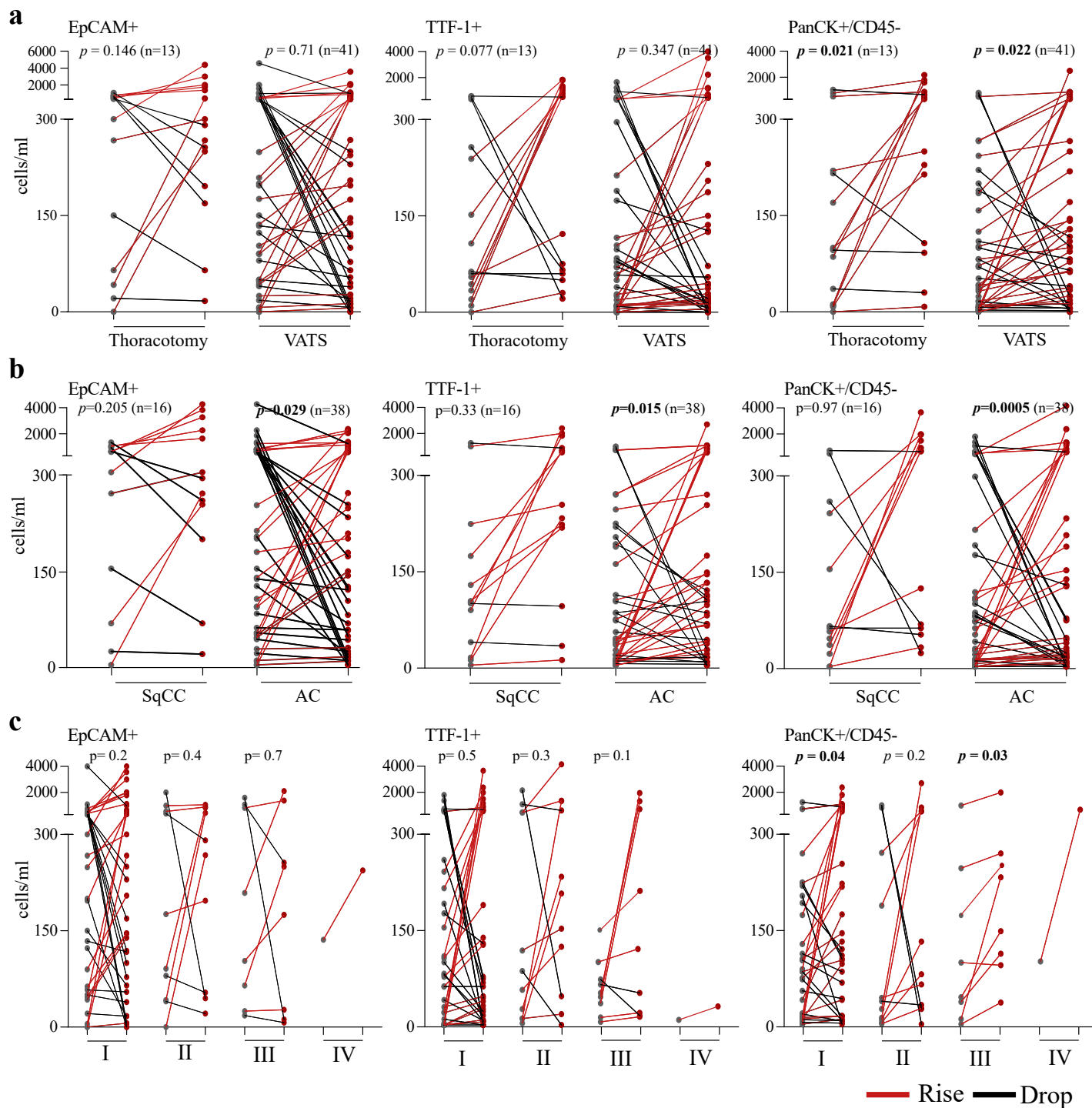

Supplement: Supplementary data [file bmjresp-2021-000917supp002.pdf]

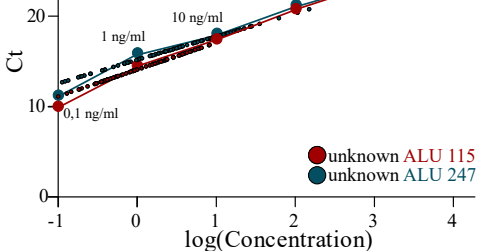

Supplement: Supplementary data [file bmjresp-2021-000917supp003.pdf]
